# Increasing facility delivery through maternity waiting homes for women living far from a health facility in rural Zambia: a quasi‐experimental study

**DOI:** 10.1111/1471-0528.16755

**Published:** 2021-06-08

**Authors:** JR Lori, ML Munro‐Kramer, H Liu, KL McGlasson, X Zhang, H Lee, T Ngoma, JL Kaiser, M Bwalya, G Musonda, I Sakala, JE Perosky, RM Fong, CJ Boyd, P Chastain, PC Rockers, DH Hamer, G Biemba, T Vian, R Bonawitz, N Lockhart, NA Scott

**Affiliations:** ^1^ University of Michigan School of Nursing Ann Arbor MI USA; ^2^ Boston University School of Public Health Boston MA USA; ^3^ Zambia Centre for Applied Health Research and Development Lusaka Zambia; ^4^ Africare/Zambia Lusaka Zambia; ^5^ Paediatric Centre of Excellence National Health Research Authority Lusaka Zambia

**Keywords:** Facility delivery, maternal health, maternity waiting homes, quasi‐experimental with partial randomisation, Zambia

## Abstract

**Objective:**

To report on the effectiveness of a standardised core Maternity Waiting Home (MWH) model to increase facility deliveries among women living >10 km from a health facility.

**Design:**

Quasi‐experimental design with partial randomisation at the cluster level.

**Setting:**

Seven rural districts in Zambia.

**Population:**

Women delivering at 40 health facilities between June 2016 and August 2018.

**Methods:**

Twenty intervention and 20 comparison sites were used to test whether MWHs increased facility delivery for women living in rural Zambia. Difference‐in‐differences (DID) methodology was used to examine the effectiveness of the core MWH model on our identified outcomes.

**Main outcome measures:**

Differences in the change from baseline to study period in the percentage of women living >10 km from a health facility who: (1) delivered at the health facility, (2) attended a postnatal care (PNC) visit and (3) were referred to a higher‐level health facility between intervention and comparison group.

**Results:**

We detected a significant difference in the percentage of deliveries at intervention facilities with the core MWH model for all women living >10 km away (DID 4.2%, 95% CI 0.6–7.6, *P* = 0.03), adolescent women (<18 years) living >10 km away (DID 18.1%, 95% CI 6.3–29.8, *P* = 0.002) and primigravida women living >10 km away (DID 9.3%, 95% CI 2.4–16.4, *P* = 0.01) and for women attending the first PNC visit (DID 17.8%, 95% CI 7.7–28, *P* < 0.001).

**Conclusion:**

The core MWH model was successful in increasing rates of facility delivery for women living >10 km from a healthcare facility, including adolescent women and primigravidas and attendance at the first PNC visit.

**Tweetable abstract:**

A core MWH model increased facility delivery for women living >10 km from a health facility including adolescents and primigravidas in Zambia.

## Tweetable abstract

A core MWH model increased facility delivery for women living >10 km from a health facility including adolescents and primigravidas in Zambia.

## Introduction

The long distances women must travel, often in labour, to reach health facilities, present one of the biggest barriers to facility delivery.^1^ Maternity waiting homes (MWHs), accommodations located near a health facility where women can stay during pregnancy and/or after birth to enable timely access to maternal and newborn healthcare, have been identified as an intervention to bridge this inequity in access caused by distance.^2^, ^3^, ^4^, ^5^

Maternity waiting homes, as a strategy to increase deliveries at health facilities with Basic Emergency Obstetric and Newborn Care (BEmONC) capacity, have been embraced as one approach to reach women who must travel long distances to deliver at a health facility.^2^ Rural health facilities are designed to be community‐based, equitable and accessible to deliver culturally appropriate and sensitive care in remote areas, where much of the population in sub‐Saharan Africa resides.^6^ Using census data from over 3800 health facilities, researchers in Ethiopia found the majority of MWHs located in rural regions.^7^

Operational models of MWHs are highly inconsistent in the materials, infrastructure and service availability between and within countries, which impacts drawing generalisable implications.^5^, ^8^, ^9^, ^10^, ^11^ Studies from several countries have found wide variation in the condition of MWHs, including lack of access to basic amenities such as electricity, toilets, cooking facilities and beds with mattresses.^7^, ^12^, ^13^ These and other studies highlight not only the importance of providing basic amenities but also the importance of community ownership for long‐term support.^13^, ^14^, ^15^

A recent meta‐analysis suggests that in low‐income countries, MWH users were 80% less likely to die than non‐users.^16^ Further analysis of these data on over 68 000 births revealed MWH use had a significant effect in reducing perinatal mortality (stillbirths, early and neonatal deaths).^17^ Two chief reasons contribute to the lack of robust evidence regarding the effectiveness of MWHs: the limited number of studies with strong methodological designs and varying operationalised models of MWHs.^2^, ^5^

The present study addresses an important research gap using a quasi‐experimental study design with partial randomisation at the cluster level to test the impact of a standardised core MWH model in rural Zambia. Our primary outcome was to determine the effectiveness of a core MWH model to increase access to facility delivery among women living far from a health facility (>10 km). Secondary outcomes included whether MWHs increased utilisation of postnatal care (PNC) and referral to the next level of care for women living >10 km from a health facility at the study sites.

## Methods

The core MWH model was developed by the Maternity Home Alliance (MHA) and is described in detail elsewhere.^18^ Briefly, the core model was co‐created with communities based on formative research in response to community standards of acceptability related to: infrastructure, equipment and supplies; policies, management and finances; and linkages and services. Examples of core model components include lighting, lockable doors, concrete flooring, formalised management structure, mechanisms for community/women’s feedback, standard operating procedures, daily check‐in by facility staff, availability of emergency transport, and provision of health education.^18^ The focus of the core model was to increase access to high‐quality obstetric services for the most vulnerable women living far from a health facility.

As part of a quasi‐experimental study design with partial randomisation at the cluster level, 20 intervention sites received the core MWH model and 20 comparison sites provided the standard of care for waiting mothers. All intervention sites received newly constructed MWHs during implementation. Standard of care for waiting mothers at facilities without a MWH included informal short stays within the health facility; a simple community‐constructed shelter at the site, where women provide their own supplies such as bedding, cooking utensils, etc.; or no dedicated space at all to wait.^19^

Two implementing partners used different methods to assign health facility sites to study arms: one used matched‐pair randomisation (10 intervention and 10 comparison) and the other a matched‐pair approach without random assignment (10 intervention and 10 comparison) due to political constraints at the district level (Figure S1).^18^ Additionally, geographic information system (GIS) techniques were used to geo‐locate and map the distance between rural villages and health facility sites in each of the catchment areas. Distances from mothers’ home villages to health facilities were calculated using ArcGIS^®^ Online (ESRI, Redlands, CA, USA). Recorded kilometer‐distances were determined as the most direct route along roads/paths between each village and their associated health facility.

### Study setting and sample

Seven districts (Chembe, Choma, Kalomo, Lundazi, Mansa, Nyimba and Pemba) in three provinces (Eastern, Luapula and Southern) were included in the study, with a total estimated population of 369 234 within‐catchment communities at all study sites. Baseline characteristics of study sites were primarily rural, with estimates of rural populations between 67% (Chembe) and 95% Lundazi.^20^ Choma/Pemba and Mansa/Chembe were administratively combined in the 2010 census. Except for Chembe, each district had at least one district hospital providing Comprehensive Emergency Obstetric and Neonatal Care (CEmONC).

The MWH intervention was examined at rural health facilities that serve women living in remote communities far from a health facility. The government of Zambia supports MWHs as one approach to increase facility delivery.^21^, ^22^ Briefly, facilities were chosen based on meeting the eligibility criteria of conducting at least 150 deliveries annually and situated ≤2 hours driving time from a facility providing CEmONC. Additionally, facilities were required to meet at least one of two sets of conditions: able to provide at least five of seven BEmONC signal functions or having at least one skilled birth attendant on staff, routinely providing active management of third stage of labour, and no stock‐outs of oxytocin or magnesium sulphate in the previous 12 months (Table S1).^18^

### Data collection

The MHA partners harmonised instruments for data collection prior to the commencement of the study. Working with their local partners, the University of Michigan collected data on the MWH sites from Chembe, Lundazi and Mansa and Boston University collected data on the MWH sites from the remaining districts. Data were extracted from Ministry of Health (MOH) registers at each of the 40 health facility sites in the study for admission, delivery, PNC and referrals for complications to the next level of care (nearest CEmONC). Additionally, data were collected through an MWH register upon admission that captured demographic data, reason for MWH stay, travel time and means of travel to MWH.

Time parameters for baseline data collection were set at 3 months prior to the opening of each individual MWH. The first MWHs opened in June 2016. Because MWHs were built and opened at various time points, time parameters for the study period included the first full month after the opening of the MWH through the end of data collection (August 2018); therefore, the study period reflects MWHs in operation between 12 and 24 months (Figure S2).

Research assistants (RAs) extracted admission, discharge and transfer data from all health facility delivery registers at all 40 sites. They also extracted data on PNC attendance and referrals from facility registers. After obtaining informed consent, admission data were collected from each woman using a MWH survey that was administered verbally by a Zambian RA in the local language.

### Data analysis

Process and outcome indicators from the two implementing partners were agreed upon by partners *a priori* and data were combined at the end of the study. The MHA partners agreed on a small set of primary and secondary outcomes prior to the intervention to answer the overarching research question: ‘does a core MWH model increase access to facility delivery for women living far from the health facility (>10 km)’. By agreeing on a limited number of indicators easily retrieved from health facility registers to examine the research question, we decreased the burden of data collection on the health system.

Descriptive analyses were performed comparing demographics across baseline and study period using Chi‐square tests for categorical variables and *t*‐tests for continuous variables. We used the difference‐in‐differences (DID) methodology to examine the effectiveness of the core MWH model on our primary and secondary outcomes. This approach adjusted for potential biases from underlying time trends and other unmeasured confounders between BEmONC facilities with MWHs (the intervention group) and BEmONC facilities without MWHs or unimproved MWHs (the comparison group).^23^ Based on data from the Saving Mothers Giving Life (SMGL) initiative Phase I and district level MOH, intervention facilities experienced a common trend in attendance for maternal and newborn services to comparison facilities until the opening of MWHs.^24^ For both groups, we calculated the proportions of women living >10 km from a health facility who came for deliveries at a BEmONC facility, attended a PNC visit at the recommended intervals (within 72 hours; 7–14 days; 6 weeks postpartum)^24^ and were referred to a higher‐level health facility in baseline and the study period. We compared the differences in the change of percentages in the intervention group versus comparison group during the study period relative to baseline (3 months prior to MWH opening) to identify associations between MWHs and outcomes.

Logistical regression was used to test the association between MWHs and the proportions of women who lived >10 km away from the facility for deliveries, PNC visits and referrals. In each model, we included two dummy variables: (1) equal to 1 for the intervention group and 0 for the comparison group and (2) equal to 1 for observations from the study period and 0 for those from baseline. We used an interaction term between these two dummy variables to perform a statistical test of the DID estimator. We then performed a risk‐adjusted model controlling for age, gravida and an indicator of whether randomisation was used in the facility assignment. We also conducted a sub‐analysis by facility assignment (randomised versus non‐randomised). All hypothesis tests were two‐sided with the level of statistical significance set to 0.05. Statistical analyses were conducted in STATA version 15.0 (StataCorp, College Station, TX, USA).

## Results

Overall, the intervention and comparison groups were similar. Delivery records from MOH registers indicated women were on average 24 years of age, having their third child, with 24–27% primigravidas. However, there was a greater number of women under age 18 years in the intervention sample than in the comparison sample at baseline (*P* = 0.01). During the course of the study period (June 2016 to 1 August 2018), 63.3% (*n* = 6622) of all women delivering at an intervention health facility used an MWH. Complete demographics are listed in Table 1.

**Table 1:** Characteristics of women delivering at health facilities at baseline and following opening of maternity waiting home (MWH) core model

	Baseline deliveries (3 months before MWHs opened)			Deliveries following opening of MWH (beginning first complete calendar month open)			Women utilising the MWH core model
	Intervention 20 sites *n* = 1570	Comparison 20 sites *n* = 1162	*P*‐value**	Intervention 20 sites *n* = 10 463	Comparison 20 sites *n* = 8081	*P*‐value**	Intervention 20 sites *n* = 6622 (63.3%)
Age, mean (SD)	24.6 (6.8)	24.7 (6.6)	0.57	24.6 (6.6)	24.7 (6.5)	0.1	24.3 (6.5)
Age <18 years, *n* (%)	183 (11.9)	103 (8.9)	0.01*	1081 (10.5)	785 (9.8)	0.13	781 (11.9)
Gravida, mean (SD)	3.2 (2.1)	3.2 (2.1)	0.64	3.2 (2.0)	3.2 (2.0)	0.95	3.2 (2.1)
Parity, mean (SD)	2.2 (2.0)	2.3 (2.0)	0.62	2.2 (2.0)	2.3 (2.0)	0.16	2.1 (2.0)
Primigravida, *n* (%)	421 (27.1)	276 (24.1)	0.08	2479 (24)	1902 (23.8)	0.7	1736 (26.3)
Grand multipara >6 pregnancies, *N* (%)	139 (8.9)	88 (7.7)	0.24	810 (7.8)	574 (7.2)	0.09	516 (7.8)
Distance from healthcare facility >10 km, *n* (%)	440 (28.3)	291 (25.1)	0.06	3185 (31)	1900 (23.6)	<0.001	2186 (38.6)

Missing data: Of total deliveries, 224 (1.05%) have missing age; 250 (1.18%) have missing gravida; 242 (1.14%) have missing parity.

*P < 0.05.

**P‐value compares intervention and comparison; two sample *t*‐test used to compare means; Chi‐square test used to compare proportions.

A total of 18 544 women delivered at an intervention or comparison health facility during the study period timeframe. Table 2 presents the absolute DID for women living >10 km away and delivering at the health facility, adolescent women (<18 years old), primigravida women and grand multipara women. The absolute DID compares facilities with the core MWH model with comparison sites. We detected a significant difference for the percentage of women delivering at a health facility living >10 km away (*P* = 0.03) in the intervention sites after the core MWH model was introduced. We also detected a significant difference for the percentage of adolescent women (*P* = 0.002) and primigravida women (*P* = 0.01) living >10 km away delivering at a health facility, with a higher percentage delivering at health facilities in the intervention group after introduction of the core MWH model. The risk‐adjusted model, controlling for age, gravida and an indicator of whether randomisation was used in the facility assignment, found no significant differences in our outcome variables for women living >10 km away.

**Table 2:** Absolute difference‐in‐differences for women living >10 km away and delivering at a health facility, *n* (%)

	Intervention sites (*n* = 20)			Comparison sites (*n* = 20)			Absolute difference‐in‐differences**			
	Baseline	Study period	Study period − baseline	Baseline	Study period	Study period − baseline	DID (95% CI)	*P*‐value	Adjusted DID (95% CI)	*P*‐value
	*n* = 1570	*n* = 10 463		*n* = 1162	*n* = 8081					
Women who delivered at a health facility, *N* (%)	440 (28.3%)	3185 (31.0%)	2.7%	291 (25.1%)	1900 (23.6%)	−1.5%	4.2% (0.6, 7.6)	0.03*	4.5% (0.8, 8.1)	0.018
Adolescent women, <18 years old (*n* = 2152)	43 (23.8)	342 (32.1)	8.30%	34 (33.0)	182 (23.2)	−9.8%	18.1% (6.3, 29.8)	0.002*	18.3% (6.5, 30.6)	0.002
Primigravida (*n* = 5078)	104 (24.9)	760 (31.0)	6.10%	75 (27.2)	456 (24.0)	−3.2%	9.3% (2.4, 16.4)	0.01*	9.5% (2.2, 16.7)	0.012
Grand multipara (*n* = 1611)	45 (32.4)	259 (32.5)	0.10%	23 (26.4)	115 (20.1)	−6.3%	6.4% (−6.5, 19.4)	0.27	6.1% (−6.7, 19.2)	0.296

* *P* < 0.05.

** The absolute difference‐in‐differences compares facilities with the core maternity waiting home (MWH) model to comparison sites.

Next, the absolute DID for women living >10 km from a health facility and attending a PNC visit within 72 hours, 7–14 days and 6 weeks postpartum and those women referred for complications to a CEmONC facility, was calculated (Table 3). There was a significant difference in women attending the first PNC visit (within 72 hours) postpartum (*P* < 0.001), with a higher percentage of change in the number of women at the intervention sites after the core MWH model was introduced. There was not a significant difference in attendance at the 7‐ to 14‐day visit (*P* = 0.414) or at the 6‐week visit (*P* = 0.612). Distance data on referrals were collected at half our study sites (*n* = 10 intervention, *n* = 10 comparison). A significant difference was noted in the proportion of women referred to the next level of care from baseline to endline in this sub‐sample (*P* = 0.023).

**Table 3:** Absolute difference‐in‐differences for women living >10 km away and attending a postnatal care (PNC) visit within 72 hours, 7–14 days and 6 weeks postpartum or referred to comprehensive emergency obstetric and neonatal care (CEmONC) facility

	Intervention sites (*n* = 20)			Comparison sites (*n* = 20)			Absolute difference‐in‐differences**	
	Baseline	Study period	Study period—baseline	Baseline	Study period	Study period − baseline	DID (95% CI)	*P*‐value
PNC visit within 72 hours postpartum	*n* = 183 43 (23.5%)	*n* = 2911 796 (27.3%)	3.8%	*n* = 142 47 (33.1%)	*n* = 2203 421 (19.1%)	−14.0%	17.8% (7.7, 28.0)	<0.001
PNC visit at 7–14 days*** postpartum	*n* = 785 230 (29.3%)	*n* = 5336 1448 (27.1%)	−2.2%	*n* = 700 155 (22.1%)	*n* = 5160 965 (18.7%)	−3.4%	1.3% (−3.4, 6)	0.414
PNC visit at 6 weeks**** postpartum	*n* = 185 43 (23.2%)	*n* = 1131 295 (26.1%)	2.8%	*n* = 154 27 (17.5%)	*n* = 848 149 (17.6%)	0.1%	2.8% (−6.5, 12.1)	0.612

	Intervention sites (*n* = 10)			Comparison sites (*n* = 10)			Absolute difference‐in‐differences**	
	Baseline	Study period	Study period − baseline	Baseline	Study period	Study period − baseline	DID (95% CI)	*P*‐value
referral to CEmONC facility	*n* = 8 1 (12.5%)	*n* = 295 77 (26.1%)	13.6%	*n* = 3 2 (66.7%)	*n* = 210 22 (10.5%)	−56.2%	69.8% (11.4, 128)	0.023

* *P* < 0.05.

** The absolute difference‐in‐differences compares facilities that have the MWH core model with comparison sites.

*** Women attending postnatal care between 4 and 14 days were included so as not to exclude women who attended their second PNC visit.

**** Women attending postnatal care between 15 and 42 days were included so as not to exclude women who attended their third PNC visit.

A sub‐analysis, by facility assignment, of women living >10 km from a health facility found a significant DID in the absolute and adjusted models for grand multipara women in the randomised group (*P* = 0.04). Alternatively, the matched‐pair without randomisation noted a significant DID in the absolute and adjusted models for women living >10 km away and delivering at the health facility (*P* = 0.002), for adolescent women (*P* = 0.001) and for primigravida women (*P* ≤ 0.001) (Table S2). Similarly, differences were noted between the randomised and non‐randomised facilities for PNC within 72 hours postpartum (*P* = 0.63 versus *P* < 0.001) (Table S3).

Using the GIS data, we calculated the travel distance for 98% of the women utilising a MWH during the study period using medians and interquartile ranges (IQR). The median distance travelled by all women utilising a MWH was 7.3 km (IQR 6.5 km), with a median length of stay of 9.0 days (IQR 19.0 days). The median length of stay based on type of care was 5.0 days (IQR 12.0 days) for antepartum care, 12.0 days (IQR 19.0 days) for those awaiting delivery, and 1.0 day (IQR 1.0 day) for those using the MWH to receive PNC. As noted in Table 4, overall 38.6% of women travelled from >10 km away, representing the largest group of women using an MWH for any reason. The median distances for each type of care received included 6.9 km (IQR 9.9 km) for antenatal care, 7.5 km (IQR 6.7 km) for those awaiting delivery, and 6.1 km (IQR 6.7 km) for those receiving postnatal care.

**Table 4:** Length of maternity waiting home (MWH) stay in days by type and distance at intervention sites

	MWH length of stay in days	Distance (km)			
	Median (IQR)	Median (IQR)	<5 km	5–10 km	>10 km
Overall (*n* = 6622)	9.0 (19.0)	7.3 (6.5)	1630 (28.8%)	1852 (32.7%)	2186 (38.6%)
By reason					
Antenatal care (*n* = 27)	5.0 (12.0)	6.9 (9.9)	10 (43.5%)	4 (17.4%)	9 (39.1%)
Awaiting delivery (*n* = 5627)	12.0 (19.0)	7.5 (6.7)	1333 (27.7%)	1613 (33.5%)	1867 (38.8%)
Postnatal care (*n* = 949)	1.0 (1.0)	6.1 (6.7)	281 (34.4%)	229 (28.1%)	306 (37.5%)

Additionally, transportation data were calculated for 97% of the women using an MWH (Figure S3). The majority of participants (82.5%) used non‐motorised means to get to the health facility, including walking, bicycle, or carried in hammock/wheelbarrow or an ox cart.

## Discussion

### Main findings

In the present study, we examined how the core MWH model can increase facility delivery, attendance at PNC visits and referral for complications to a CEmONC facility for women living >10 km away from a health facility. Overall, this study found the core MWH model was successful in increasing facility delivery and attendance at the first PNC visit for women living >10 km from a healthcare facility. The core MWH model also increased the percentage of women <18 years old and primigravida women living >10 km away accessing health facilities for deliveries. There was no significant difference for the percentage of women attending a PNC visit at 7–14 days or 6 weeks postpartum living >10 km away. A significant difference was found among women referred to a CEmONC facility, but there are limitations to this finding due to the small sample size at baseline.

### Strength and limitations

This study has several strengths, including a large sample of women living in rural, remote areas of Zambia and the use of selection criteria to match comparison and intervention sites.^18^ Additionally, the harmonisation of indicators prior to the start of data collection ensured that partners used the same definitions and measured similar outcomes.

There are several limitations that constrain interpretation of the findings. First, implementing partners used different methods to select and assign health facility sites to study arms. In four districts, one partner randomly assigned health facilities to receive the MWH intervention, whereas in three districts, the second partner used input from district health teams and purposively sampled from eligible rural health facilities.^18^ Due to lack of randomisation, selection bias may have been introduced into the study. In our sensitivity analysis, we conducted a risk‐adjusted model and did not find a significant difference between facilities randomised and those not randomised. This may be a reflection of the strict criteria established for inclusion in the study. We acknowledge that it is possible there are inherent differences between the comparison and intervention sites that cannot be fully adjusted. Secondly, the study was conducted in districts where the SMGL initiative had implemented evidence‐based interventions to reduce maternal and newborn mortality, including improving the quality of BEmONC services while improving access and demand.^25^ However, these SMGL districts were purposively chosen to ensure adequate quality of care if the intervention increased access and demand. Additionally, distance data for women referred to a CEmONC facility was only collected at half our study sites, leading to small numbers at baseline and threatening the validity of these results. Finally, MOH facility registers were used for collection of various data. Data were entered into these registers by the nurse or midwife on duty. Although each health facility was issued standard data collection registers with definitions for each cell, there was the chance of varying interpretation by the recorder. To address this, we conducted training at each site with nurses and midwives as well as field staff who worked with nurses and midwives to ensure accuracy of the data.

### Interpretation

Overall, this study found the core MWH model was successful in reaching women with historically low rates of facility delivery and attendance at the first PNC visit—those living >10 km from a healthcare facility. Data on utilisation of maternal and newborn care from five East African countries suggests that greater geographical inaccessibility (often defined as >10 km from a health facility) contributes to lower rates of receiving recommended antenatal care, delivering at a facility with a skilled birth attendant, and obtaining PNC.^26^ The core MWH model provided access to this population regardless of how they initially reached the MWH, via motorised or non‐motorised transportation.

The postnatal period, the days and weeks following delivery, represent a critical phase in women and newborn’s lives. The World Health Organization recommends at least three postnatal contacts following delivery for all mothers and newborns.^24^ Pooled analysis of nine studies from low‐ and middle‐income countries found that 75% of newborn deaths occur in the first week of life (74.3%), with the highest number of deaths in the first week during the first 3 days of life (37.6%).^24^ The core MWH model increased the proportion of women attending this essential PNC visit within the first 72 hours post‐delivery.

While a statistically significant difference was found in the average referral rate between the intervention and comparison sites, the small number of referrals at baseline contributes to the lack of robustness of these data. Our study was intentionally conducted in districts where SMGL had previously been implemented to provide quality services for women choosing facility delivery. Prior to the beginning of this study, all sites received upgrading of services through the SMGL initiative, including improving communication and transportation systems.^27^ Further research is needed to assess the impact of MWHs on referral patterns for women living >10 km from a health facility.

There may be several explanations for the differences for women living >10 km from a health facility noted at a sub‐analysis level. Temporal variations may have occurred in collection of baseline data. Baseline data were collected in the non‐randomised communities 3 months prior to baseline data in the randomised communities and therefore a longer timeframe for study period data collection occurred in the non‐randomised communities. Additionally, in the non‐randomised communities, site selection was driven by the district ministry, who expressed concern that community fatigue due to large numbers of projects and research activities in the area would affect implementation.^18^

In addition to increasing access for all women at geographical risk, the core MWH model also increased the percentage of adolescent women (<18 years old) and primigravida women living >10 km away accessing health facilities for deliveries. Adolescents are known to be at greater risk for maternal morbidity and mortality due to biological and socio‐cultural factors.^28^ The government of Zambia specifically recommends that all adolescent pregnancies, primigravidas and grand multiparas should deliver at a health facility due to increased risk for maternal morbidity and mortality related to age and pregnancy status.^29^

A persistent decline was seen in almost all our outcome variables from the baseline‐study period in the comparison communities. It is possible that women chose to deliver at facilities with MWHs. Several studies have shown women regularly bypass clinics in search of quality services.^30^, ^31^

Past research has noted there are numerous barriers to the use of MWHs once they are constructed, and some studies have seen minimal use and sustainability.^32^, ^33^, ^34^ Maternity waiting homes in Guatemala and Ethiopia were not used due to lack of knowledge and community awareness about the homes and limited provision of culturally appropriate care.^34^, ^35^ A qualitative thematic synthesis of 29 studies from 17 countries additionally noted poor utilisation due to inadequate structures, absence of community involvement in the design and upkeep, as well as culturally inappropriate care and lack of knowledge and acceptance by women and community members.^5^ Alternatively, the core MWH model implemented in this study incorporated many of the facilitators identified in past research, including no cost to stay, community involvement, awareness raising and integration of culturally appropriate practices to ensure uptake and sustainability.^4^, ^5^, ^12^, ^13^ Early harmonisation of indicators ensured that MHA partners used the same definitions and measured similar outcomes. This allowed for comparisons using all partner data and is essential to ensure that large‐scale data obtained using a quasi‐experimental design are comparable across sites. This methodology addresses many of the problems noted in the literature that have led to mixed and inconclusive results regarding the outcomes and effectiveness of MWHs.^2^, ^5^, ^16^

## Conclusion

This study is one of the first to examine the impact of an MWH intervention to increase access to reproductive health services for women living >10 km from a rural health facility. Results of this study indicate that a community‐driven, entrepreneurial core MWH model is effective at increasing facility delivery for women living far from the health facility (>10 km), especially primigravidas and women <18 years old. The core MWH model also significantly increased attendance at the first PNC visit for women living >10 km from the health facility, a critical time in the lives of women and newborns. Maternity waiting homes are one strategy to improve access to facility delivery for women living a great distance from a healthcare facility.

### Disclosure of interests

None declared. Completed disclosure of interests forms are available to view online as supporting information.

### Contribution to authorship

JRL, CJB, DHH, NAS designed the study and data collection instruments. TN, JLK, MB, GM, JEP collected data. JRL, MLMK, HL, KLM, XZ, PK, PCR, NL, NAS managed and conducted data analysis. JRL, MLMK, HL, KLM, XZ, HL, TN, JLK, MB, GM, IS, JEP, RMF, CJB, PC, PCR, DHH, GB, TV, RB, NL, NAS contributed to the development of the manuscript. All authors reviewed and approved the final version of the manuscript.

### Details of ethics approval

Ethical approvals were obtained from the University of Michigan (Ref No. HUM00110404, Date of Approval 18 January 2016) and Boston University Institutional Review Boards (Ref No. H‐34526, Date of Approval 12 January 2016) as well as ERES Converge (Where Research, Ethics, and Science Converge) IRB (Ref No. 00005948, Date of Approval 14 December 2015), a private research ethics board in Zambia governed by the National Health Research Ethics Committee. We also obtained approval to proceed with the study from the Zambia National Health Research Authority, which is responsible for oversight of all research conducted in that country.

### Funding

This programme was developed and is being implemented in collaboration with *Merck for Mothers*, Merck’s 10‐year, US$500‐million initiative to help create a world where no woman dies giving life. *Merck for Mothers* is known as *MSD for Mothers* outside the USA and Canada (MRK 1846‐06500.COL). The development of this article was additionally supported in part by the Bill & Melinda Gates Foundation (OPP1130334) www.gatesfoundation.org/How‐We‐Work/Quick‐Links/Grants‐Database/Grants/2015/06/OPP1130334 and The ELMA Foundation (ELMA‐15‐F0010) www.elmaphilanthropies.org/the‐elma‐foundation/. The funders had no role in study design, data collection and analysis, decision to publish or preparation of the manuscript. The content is solely the responsibility of the authors and does not necessarily reflect positions or policies of Merck, the Bill & Melinda Gates Foundation or The ELMA Foundation. Funding for Online Open publication was supported by Chronos Support through the Bill & Melinda Gates Foundation.

## Supporting information


**Figure S1.** Study sites and participants by randomisation and non‐randomisation.

**Figure S2.** Study timeline.

**Figure S3.** Comparison of non‐motorised versus motorised transportation by time in hours.

**Table S1.** Health facilities meeting eligibility criteria.
**Table S2.** Absolute difference‐in‐differences for women living >10 km away group by sites; *n* (%).
**Table S3.** Absolute difference‐in‐differences for women attending a postnatal care (PNC) visit within 72 hours, 7–14 days and 6 weeks postpartum, by site.

Supplementary Material

## Data Availability

The data that support the findings of this study are available from the corresponding author upon reasonable request.

## Authors’ roles in the reported study

Jody R. Lori^1^, PI
Michelle L. Munro‐Kramer^1^, co‐I
Haiyin Liu^1^, data analyst
Kathleen L. McGlasson^2^, data analyst
Xingyu Zhang^1^, data analyst
HaEun Lee^1^, data management
Thandiwe Ngoma^3^, co‐PI
Jeanette L. Kaiser^2^, co‐I
Misheck Bwalya^3^, co‐I
Gertrude Musonda^4^, co‐I
Isaac Sakala^4^, co‐PI
Joseph E. Perosky^1^, data management
Rachel M. Fong^2^,
Carol J. Boyd^1^, co‐I
Parker Chastain^2^, co‐I
Peter C. Rockers^2^, co‐I
Davidson H. Hamer^2^, co‐I
Godfrey Biemba^5^, co‐I
Taryn Vian^2^, co‐I
Rachael Bonawitz^2^, co‐I
Nancy Lockhart^1^, data management
Nancy A. Scott^2^ PI

## References

[1] Dimbuene Z , Amo‐Adjei J , Amugsi D , Mumah J , Izugbara C , Beguy D . Women’s education and utilization of maternal health services in Africa: a multi‐country and socioeconomic status analysis. J Biosoc Sci 2015;50:725–48. 10.1017/S0021932017000505 29103388

[2] Lonkhuijzen L , Stekelenburg J , Roosmalen J . Maternity waiting facilities for improving maternal and neonatal outcome in low‐resource countries. Cochrane Database Syst Rev 2014;(10):CD006759. 10.1002/14651858.CD006759. PMC4098659 23076927

[3] World Health Organization . Maternity Waiting Homes: A Review of Experiences. Geneva: World Health Organization, Maternal and Newborn Health Safe Motherhood Unit, Division of Reproductive Health; 1996.

[4] Lori J , Munro‐Kramer M , Mdluli E , Musonda G , Boyd C . Developing a community driven sustainable model of maternity waiting homes for rural Zambia. Midwifery 2016;41:89–95. 27571773 10.1016/j.midw.2016.08.005

[5] Penn‐Kekana L , Pereira S , Hussein J , Bontogon H , Chersich M , Munjanja S , et al. Understanding the implementation of maternity waiting homes in low‐ and middle‐income countries: a qualitative thematic synthesis. BMC Pregnancy Childbirth 2017;17:269. 28854880 10.1186/s12884-017-1444-z PMC5577673

[6] World Bank . Rural population (% of total populations) sub‐Saharan Africa. 2018. [https://data.worldbank.org/indicator/SP.RUR.TOTL.ZS?locations=ZG]. Accessed 16 December 2020.

[7] Tiruneh GT , Getu YN , Abdukie MA , Eba GG , Keyes M , Bailey PE . Distribution of maternity waiting homes and their correlation with perinatal mortality and direct obstetric complication rates in Ethiopia. BMC Pregnancy Childbirth 2019;19:214. 31238909 10.1186/s12884-019-2356-x PMC6593553

[8] Bergen N , Abebe L , Asfaw S , Kiros G , Kulkarni MA , Mamo A , et al. Maternity waiting areas—serving all women? Barriers and enablers of an equity‐oriented maternal health intervention in Jimma Zone, Ethiopia. Glob Public Health 2019;14:1509–23. 30905270 10.1080/17441692.2019.1597142

[9] Gaym A , Pearson L , Soe K . Maternity waiting homes in Ethiopia—three decades experience. Ethiop Med J 2012;50:209–19. 23409404

[10] Chandramohan D , Cutts F , Chandra R . Effects of a maternity waiting home on adverse maternal outcomes and the validity of antenatal risk screening. Int J Gynaecol Obstet 1994;46:279–84. 7805996 10.1016/0020-7292(94)90406-5

[11] Kelly J , Kohls E , Poovan P , Schiffer R , Redito A , Winter H , et al. The role of a maternity waiting area (MWA) in reducing maternal mortality and stillbirths in high‐risk women in rural Ethiopia. BJOG 2010;117:1377–83. 20670302 10.1111/j.1471-0528.2010.02669.x

[12] Scott NA , Vian T , Kaiser JL , Ngoma T , Mataka K , Henry EG , et al. Listening to the community: using formative research to strengthen maternity waiting homes in Zambia. PLoS One 2018;13:e0194535. 29543884 10.1371/journal.pone.0194535 PMC5854412

[13] Lori JR , Perosky JE , Rominski S , Munro‐Kramer ML , Cooper F , Kofa A , et al. Maternity waiting homes in Liberia: results of a country wide mulit‐sector scale‐up. PLoS One 2020;15:e0234785. 32574182 10.1371/journal.pone.0234785 PMC7310707

[14] Fontanet CP , Fong RM , Kaiser JL , Bwalya M , Ngoma T , Vian T , et al. A qualitative exploration of community ownership of a maternity waiting home model in rural Zambia. Glob Health Sci Pract 2020;8:344–57. 33008852 10.9745/GHSP-D-20-00136 PMC7541113

[15] Satti H , McLaughlin MM , Seung KJ . The role of maternity waiting homes as part of a comprehensive maternal mortality reduction strategy in Lesotho. PIH Rep 2013;1:1–9.

[16] Dadi T , Bekele B , Kasaye H , Nigussie T . Role of maternity waiting homes in the reduction of maternal death and stillbirth in developing countries and its contribution for maternal death reduction in Ethiopia: a systematic review and meta‐analysis. BMC Health Serv Res 2018;18:748. 30285757 10.1186/s12913-018-3559-y PMC6167854

[17] Bekele B , Dadi T , Tesfaye T . The significant association between maternity waiting homes utilization and perinatal mortality in Africa: systematic review and meta‐analysis. BMC Res Notes 2019;12:13. 30642355 10.1186/s13104-019-4056-z PMC6332606

[18] Scott NA , Kaiser JL , Vian T , Bonawitz R , Fong RM , Ngoma T , et al. Impact of maternity waiting homes on facility delivery among remote households in Zambia: Protocol for a quasiexperimental, mixed‐methods study. BMJ Open 2018;8:e022224. 10.1136/bmjopen-2018-022224 PMC6089313 30099401

[19] Henry EG , Semrau K , Hamer DH , Vian T , Nambao M , Mataka K , et al. The influence of quality maternity waiting homes on utilization of facilities for delivery in rural Zambia. Reprod Health 2017;14:68. 28558800 10.1186/s12978-017-0328-z PMC5450262

[20] Central Statistical Office Zambia . 2010 Census of population and housing – Southern Province analytical report. [www.zamstats.gov.zm/phocadownload/2010_Census/2010_Census_Analytical_Reports/Southern%20Province%20Analytical%20Report%20‐%202010%20Census.pdf]. Accessed 20 December 2020.

[21] Republic of Zambia Ministry of Health . Roadmap for accelerating reduction of maternal, newborn and child mortality, 2013–2016. 2016, pp. 2013–6. [https://bettercarenetwork.org/sites/default/files/Roadmap%20for%20Accelerating%20Reduction%20of%20Maternal%2C%20Newborn%20and%20Child%20Mortality%20‐%20Zambia%202013‐2016.pdf]. Accessed 12 December 2020.

[22] Ministry of Health (MOH) . Zambia National Health Strategic Plan 2017–2021. 2017. [https://www.moh.gov.zm/?wpfb_dl=87]. Accessed 12 December 2020.

[23] Dimick JB , Ryan AM . Methods for evaluating changes in health care policy: the difference‐in‐differences approach. JAMA 2014;312:2401–2. 25490331 10.1001/jama.2014.16153

[24] World Health Organization . WHO recommendation on postnatal care of the mother and newborn. 2014. [https://apps.who.int/iris/bitstream/handle/10665/97603/9789241506649_eng.pdf?sequence=1]. Accessed 12 December 2020. 24624481

[25] Centers for Disease Control and Prevention . Saving Mothers, Giving Life: Monitoring and Evaluation Overview, Phase 1 Report. Atlanta: Centers for Disease Control and Prevention, U.S. Dept of Health and Human Services; 2014. [www.cdc.gov/reproductivehealth/global/publications/pdfs/MonitoringandEvaluationOverview.pdf]. Accessed 11 November 2020.

[26] Ruktanonchai CW , Ruktanonchai NW , Nove A , Lopes S , Pezzulo C , Bosco C , et al. Equality in maternal and newborn health: modelling geographic disparities in utilisation of care in five East African countries. PLoS One 2016;11:e0162006. 27561009 10.1371/journal.pone.0162006 PMC4999282

[27] Ngoma T , Asiimwe AR , Mukasa J , Binzen S , Serbanescu F , Henry EG , et al. Addressing the second delay in saving mothers, giving life districts in Uganda and Zambia: reaching appropriate maternal care in a timely manner. Glob Health Sci Pract 2019;7 (Suppl 1):S68–84. 30867210 10.9745/GHSP-D-18-00367 PMC6519669

[28] Ganchimeg T , Ota E , Morisaki N , Laopaiboon M , Lumbiganon P , Zhang J , et al. Pregnancy and childbirth outcomes among adolescent mothers: a World Health Organization multicountry study. BJOG 2014;121:40–8. 10.1111/1471-0528.12630 24641534

[29] Republic of Zambia, Ministry of Health . Reproductive, maternal, newborn, child and adolescent health and nutrition communication and advocacy strategy 2018–2021. [https://www.moh.gov.zm/?wpfb_dl=111]. Accessed 11 November 2020.

[30] Kruk ME , Paczkowski M , Mbaruku G , de Pinho H , Galea S . Women’s preferences for place of delivery in rural Tanzania: a population‐based discrete choice experiment. Am J Public Health 2009;99:1666–72. 19608959 10.2105/AJPH.2008.146209 PMC2724466

[31] Montagu D , Sudhinaraset M , Diamond‐Smith N , Campbell O , Gabrysch S , Freedman L , et al. Where women go to deliver: understanding the changing landscape of childbirth in Africa and Asia. Health Policy Plan 2017;32:1146–52. 28541422 10.1093/heapol/czx060 PMC5886217

[32] Eckermann E , Deodato G . Maternity waiting homes in Southern Lao PDR: the unique ‘silk home’. J Obstet Gynaecol Res 2008;34:767–75. 18834334 10.1111/j.1447-0756.2008.00924.x

[33] García Prado A , Cortez R . Maternity waiting homes and institutional birth in Nicaragua: policy options and strategic implications. Int J Health Plann Manage 2012;27:150–66. 22052420 10.1002/hpm.1107

[34] Ruiz MJ , van Dijk MG , Berdichevsky K , Munguía A , Burks C , García SG . Barriers to the use of maternity waiting homes in indigenous regions of Guatemala: a study of users’ and community members’ perceptions. Cult Health Sex 2013;15:205–18. 23234509 10.1080/13691058.2012.751128

[35] Vermeiden T , Braat F , Medhin G , Gaym A , van den Akker T , Stekelengurg J . Factors associated with intended use of a maternity waiting home in Sothern Ethiopia: a community‐based cross‐sectional study. BMC Pregnancy Childbirth 2018;18:38. 29351786 10.1186/s12884-018-1670-z PMC5775531

