# Supplementary material for: Increasing facility delivery through maternity waiting homes for women living far from a health facility in rural Zambia: a quasi‐experimental study
Source: BJOG. 2021 Jun 8;128(11):1804–12. doi: 10.1111/1471-0528.16755 (PMC8518771; doi:10.1111/1471-0528.16755)
Supplement: Supplementary file 1 — Figure S1. Study sites and participants by randomisation and non‐randomisation. [file BJO-128-1804-s003.docx]

**Figure S1.** Study Sites and Participants by Randomization and Non-Randomization

Total Deliveries

Study period = 18,544

Non-randomized sites (9,102)

Intervention (5,307)

Comparison (3,795)

>10Km (1,508)

Stayed at MWH (838)

<10km (3,793)

>10Km (935)

<10Km (2,854)

Stayed at MWH (2,010)

Randomized sites (9,442)

Intervention (5,156)

Comparison (4,286)

>10Km (1,677)

Stayed at MWH (1,348)

<10Km (3,306)

>10Km (965)

<10Km (3,288)

Stayed at MWH (1,677)
