# Supplementary material for: Increasing facility delivery through maternity waiting homes for women living far from a health facility in rural Zambia: a quasi‐experimental study
Source: BJOG. 2021 Jun 8;128(11):1804–12. doi: 10.1111/1471-0528.16755 (PMC8518771; doi:10.1111/1471-0528.16755)
Supplement: Supplementary file 2 — Figure S2. Study timeline. [file BJO-128-1804-s002.docx]

**Figure S2** Study timeline.

Study Period

Data collection

Randomized sites

Study Period

Data collection

Non-randomized sites

2015 - 2016

June 2016

June - August 2016

July 2017

March - May 2016

August 2018

September 2016
