# Supplementary material for: Increasing facility delivery through maternity waiting homes for women living far from a health facility in rural Zambia: a quasi‐experimental study
Source: BJOG. 2021 Jun 8;128(11):1804–12. doi: 10.1111/1471-0528.16755 (PMC8518771; doi:10.1111/1471-0528.16755)
Supplement: Supplementary file 3 — Figure S3. Comparison of non‐motorised versus motorised transportation by time in hours. [file BJO-128-1804-s005.docx]

**Figure S3.** Comparison of non-motorized versus motorized transportation by time in hours.
