# Supplementary material for: Increasing facility delivery through maternity waiting homes for women living far from a health facility in rural Zambia: a quasi‐experimental study
Source: BJOG. 2021 Jun 8;128(11):1804–12. doi: 10.1111/1471-0528.16755 (PMC8518771; doi:10.1111/1471-0528.16755)
Supplement: Supplementary file 4 — Table S1. Health facilities meeting eligibility criteria. Table S2. Absolute difference‐in‐differences for women living >10 km away group by sites; n (%). Table S3. Absolute difference‐in‐differences for women attending a postnatal care (PNC) visit within 72 hours, 7–14 days and 6 weeks postpartum, by site. [file BJO-128-1804-s001.docx]

**Table S1.** Health Facilities Meeting Eligibility Criteria

|  | Kalomo, Nyimba, Choma/Pemba | Lundazi, Mansa/Chembe |
| --- | --- | --- |
| Facilities ≤ 2 hours from CEmONC *and* min of 150 deliveries/year | 36 | 29 |
| Met one of two eligibility criteria | 22 | 22 |
| Selected for study | 20 | 20 |

**Table S2.** Absolute difference-in-differences for women living >10km away group by sites, N(%).

|  | **Intervention Sites** | | | **Comparison Sites** | | | **Absolute Difference-in-Differences^** | | | |
| --- | --- | --- | --- | --- | --- | --- | --- | --- | --- | --- |
|  | **Baseline** | **Study Period** | **Study period – Baseline** | **Baseline** | **Study Period** | **Study period – Baseline** | **DID**  **(95% CI)** | **p-value** | **Adjusted DID (95% CI)** | **p-value** |
|  |  |  |  |  |  |  |  |  |  |  |
| **Randomized Sites** | N=846 | N=5156 |  | N=638 | N=4286 |  |  |  |  |  |
| Women who delivered at a health facility (N=10,926) | 286(34.2) | 1677(33.7) | -0.50 | 156(24.5) | 965(22.7) | -1.8 | 1.3% (-3.7, 6.2) | 0.5539 | 1.6% (-3.4, 6.6) | 0.47 |
| Adolescent Women, <18 years old (N=1240) | 29(29.3) | 207(34.9) | 5.6 | 14(23.3) | 97(20.6) | -2.7 | 8.3% (-6.6, 23.2) | 0.3026 | 8.8% (-6.2, 23.8) | 0.275 |
| Primigravida (N=2559) | 73(34.8) | 412(34.2) | -0.6 | 31(22.0) | 220(22.7) | 0.7 | -1.3% (-11.4, 8.9) | 0.8116 | -0.9% (-11.1, 9.2) | 0.858 |
| Grand Multipara (N=907) | 28(30.4) | 183(38.5) | 8.1 | 12(26.1) | 46(16.6) | -9.5 | 17.6% (5.9, 34.5) | 0.036 | 17.1% (1.1, 34.3) | 0.042 |
|  |  |  |  |  |  |  |  |  |  |  |
| **Non Randomized Sites** | N=724 | N=5307 |  | N=524 | N=3795 |  |  |  |  |  |
| Women who delivered at a health facility, (N=10,350) | 154(21.3) | 1508(28.5) | 7.20 | 135(25.9) | 935(24.7) | -1.2 | 8.4% (3.1, 13.4) | 0.0021 | 8.3% (3.1, 13.5) | 0.002 |
| Adolescent Women, <18 years old (N=912) | 14(17.1) | 135(28.5) | 11.4 | 20(46.5) | 85(27.2) | -19.3 | 30.8% (12.6, 50.0) | 0.0009 | 30.5% (12.3, 48.7) | 0.001 |
| Primigravida (N=2519) | 31(15.0) | 348(28.0) | 13.0 | 44(32.6) | 236(25.5) | -7.1 | 20.1% (10.1, 30.1) | <.0001 | 20.0% (10.0, 30.0) | <0.001 |
| Grand Multipara (N=704) | 17(36.2) | 76(23.7) | -12.5 | 11(26.8) | 69(23.5) | -3.3 | -9.2% (-29.5, 11.3) | 0.399 | -9.0% (-29.6, 11.4) | 0.403 |

^The absolute difference in differences compares facilities with the core Maternity Waiting Home (MWH) Model to Comparison Sites

*p<0.05

**Table S3.** Absolute difference-in-differences for women living >10km away attending a postnatal care (PNC) visit within 72 hours, 7-14 days and 6 weeks postpartum by sites.

|  | **Intervention Sites** | | | **Comparison Sites** | | | **Absolute Difference-in-Differences^** | |
| --- | --- | --- | --- | --- | --- | --- | --- | --- |
|  | **Baseline** | **Study Period** | **Study period – Baseline** | **Baseline** | **Study Period** | **Study period – Baseline** | **DID**  **(95% CI)** | **p-value** |
|  |  |  |  |  |  |  |  |  |
| **Randomized Sites** |  |  |  |  |  |  |  |  |
| PNC visit within 72 hours postpartum | N=16  2 (12.5%) | N=532  163 (30.6%) | 18.1% | N=29  3(10.3%) | N=353  64 (18.1%) | 7.8% | 10.4% (-10.1, 30.8) | 0.628 |
| PNC visit at 7-14 days postpartum** | N = 481  158 (32.9%) | N = 3073  898 (29.2%) | -3.6% | N = 398  78 (19.6%) | N = 2768  471 (17%) | -2.6% | -1% (-7.1%, 5.1%) | 0.985 |
| PNC visit at 6 weeks (15-42 days) postpartum*** | N = 47  13 (27.7%) | N = 423  146 (34.5%) | 6.9% | N = 22  3 (13.6%) | N = 217  32 (14.7%) | 1.1% | 5.8% (-14.5%, 26%) | 0.754 |
|  |  |  |  |  |  |  |  |  |
|  |  |  |  |  |  |  |  |  |
| **Non-randomized Sites** |  |  |  |  |  |  |  |  |
| PNC visit within 72 hours postpartum | N=167  41 (24.5%) | N=2379  633 (26.6%) | 2.1% | N=113  44 (38.9%) | N=1850  357 (19.3%) | -19.6% | 21.7% (10.3, 33.1) | <0.001 |
| PNC visit at 7-14 days postpartum** | N = 304  72 (23.7%) | N = 2263  550 (24.3%) | 0.6% | N = 302  77 (25.5%) | N = 2392  494 (20.6%) | -4.9% | 5.5% (-1.8%, 12.7%) | 0.127 |
| PNC visit at 6 weeks (15-42 days) postpartum*** | N = 138  30 (21.7%) | N = 708  149 (21.1%) | -0.7% | N = 132  24 (18.2%) | N = 631  117 (18.5)% | 0.4% | -1.1% (-11.5%, 9.4%) | 0.846 |
|  |  |  |  |  |  |  |  |  |

^The absolute difference in differences compares facilities with the MWH Core Model to Comparison Sites

*p<0.05

**Women attending postnatal care between 4-14 days were included to not exclude women who attended their second PNC visit

***Women attending postnatal care between 15-42 days were included to not exclude women who attended their third PNC visit
